# Genetic Manipulation in *Sporothrix* Species: Molecular Tools, Challenges, and Applications

**DOI:** 10.3390/jof12010061

**Published:** 2026-01-13

**Authors:** Mafalda Barros, Matheus Tavares, Ricardo Silvestre, Roberta Peres da Silva, Fernando Rodrigues

**Affiliations:** 1 Life and Health Sciences Research Institute (ICVS), School of Medicine, University of Minho, 4710-057 Braga, Portugal; id11855@alunos.uminho.pt (M.B.); id11290@alunos.uminho.pt (M.T.); ricardosilvestre@med.uminho.pt (R.S.); 2 ICVS/3B’s-PT Government Associate Laboratory, 4806-909 Braga, Portugal; 3 Department of Microbiology, Immunology and Parasitology, Paulista School of Medicine, Federal University of São Paulo (UNIFESP), São Paulo 04023-062, Brazil; roberta.peres@unifesp.br

**Keywords:** *Sporothrix brasiliensis*, thermal dimorphism, virulence factors, genetic tools, fungal transformation, CRISPR/Cas9

## Abstract

*Sporothrix* species are thermally dimorphic fungi responsible for sporotrichosis, a globally prevalent subcutaneous mycosis and an emerging zoonotic threat, particularly in South America. The high virulence of *Sporothrix brasiliensis* and its efficient transmission from cats to humans have intensified recent outbreaks, underscoring the importance of understanding the pathogenic mechanisms. While several putative virulence factors have been identified, such as melanin production, cell wall remodeling, extracellular vesicles, and thermotolerance, functional studies remain hampered by limited molecular tools. Recent advances, including random mutagenesis, protoplast-mediated transformation, *Agrobacterium tumefaciens*-mediated transformation, RNA interference and CRISPR/Cas9-based genome editing, are changing this landscape. These methods have enabled the functional validation of key virulence factors and the investigation of gene function in both environmental and clinical strains. In this review, we summarize the genetic toolbox available for *Sporothrix*, outline current challenges, and discuss how these strategies are reshaping the study of fungal virulence and host–pathogen interactions.

## 1. *Sporothrix* Species and Sporotrichosis

Sporotrichosis is a subcutaneous disease caused by thermally dimorphic fungi of the genus *Sporothrix*, which are commonly found in soil and decaying organic matter [1,2]. Phylogenetic analyses have revealed that *Sporothrix schenckii*, historically considered the only etiological agent, represents a cryptic species complex comprising several clinically relevant species, namely *S. schenckii* sensu stricto, *S. brasiliensis*, *S. globosa*, and *S. luriei* [1,3], which form a distinct lineage from plant-associated *Sporothrix* species.

Thermal dimorphism represents a key virulence attribute in pathogenic *Sporothrix* species. Under environmental conditions (25 °C to 27 °C), the fungus grows as conidium-producing mycelia, whereas exposure to host physiological temperatures (35 °C to 37 °C) triggers the transition to the parasitic yeast phase. This transition is essential for establishing an infection and disseminating it to different tissues [2]. Despite sharing this conserved trait, each species exhibits different pathogenic potential, largely influenced by host–pathogen interactions and immune evasion strategies [4].

Pathogenic *Sporothrix* species differ in both transmission patterns and geographic distribution, reflecting ecological adaptation and virulence potential. *S. schenckii*, the primary agent of sporotrichosis in most endemic regions, is widely distributed worldwide and predominantly transmitted sapronotically from environmental sources, particularly in tropical and subtropical areas [2]. In contrast, *S. brasiliensis* is largely restricted to South America and has emerged as a highly virulent zoonotic pathogen. Domestic cats serve as efficient reservoirs and transmitters of the fungus to other animals and humans through scratches, bites, or contact with infected secretions. This shift from environmental to animal-associated transmission leads to more severe and atypical clinical manifestations, including extracutaneous disease, in both immunocompetent and immunocompromised hosts [5,6,7,8]. *S. globosa* predominates in parts of Asia, illustrating the species-specific geographic structuring of sporotrichosis. The emergence of *S. brasiliensis* highlights the link between transmission mode, epidemic potential, and pathogenic fitness.

Virulence factors are microbial components, including structural molecules and specialized strategies, that promote host colonization by facilitating adhesion, invasion, nutrient acquisition, immune evasion, and survival within host tissues. These factors are often dispensable for saprophytic growth but are essential for successful pathogenesis. In pathogenic fungi, virulence is best understood in the context of dynamic host–pathogen interactions, and advances in genomics, transcriptomics, and proteomics have enabled the identification of conserved and species-specific virulence determinants.

Among medically relevant fungi, canonical virulence traits of *Sporothrix* species include thermal dimorphism and thermotolerance, remodeling of the fungal cell wall, expression of heat shock proteins (HSPs), production of protective pigments such as melanin, secretion of hydrolytic enzymes, resistance to host-derived stresses, and the ability to form biofilms [9,10]. Although comprehensive reviews of *Sporothrix* virulence have been published elsewhere [9,11,12], the following section summarizes the principal factors identified to date and highlights how recent molecular approaches have enabled more in-depth functional characterization.

## 2. Virulence Factors and Sporothrix Species Pathogenicity

The mycelium-to-yeast transition of *Sporothrix* species is essential for pathogenicity. This morphological switch is known to be regulated by complex signaling pathways, including those involving calcium signaling and the DRK1 (Dimorphism-Regulating Kinase 1). In *S. schenckii*, calcium uptake stimulates the dimorphic transition by activating Ca^2+^/calmodulin-dependent protein kinases (CaMKs) via interaction with calmodulin (CaM). The kinase Sscmk1, a central CaMK in *S. schenckii*, is essential for both the yeast-to-mycelium switch and proper progression through the yeast cell cycle [13,14].

The fungal cell wall plays a key role in the virulence of *Sporothrix* species, mediating host adhesion, immune evasion, and infection. Its composition is dynamic and influenced by environmental conditions, such as growth media and nutrient availability, affecting cell wall architecture and virulence potential [15]. Nutrient availability modulates fungal metabolic pathways and influences cell wall remodeling, thereby contributing to differences in pathogenic potential. Consequently, inoculum preparation conditions in experimental infection models represent an important determinant of virulence. The inoculum of *S. brasiliensis* and *S. schenckii* prepared under carbon- or nitrogen-limited conditions displays reduced virulence due to remodeling in cell wall architecture and composition [16,17].

Villalobos-Duno et al. evaluated the composition of polysaccharides of the pathogenic yeast form of five *Sporothrix* strains, two of *S. brasiliensis* and three of *S. schenckii*, with differences in virulence profile [17]. They reported a higher chitin content in the cell wall of all *S. brasiliensis* strains tested when compared to the other *Sporothrix* species. Remarkably, these authors found a mathematical expression that establishes the positive relation between the cell wall rhamnose-to-β-glucan ratio with the virulence in *Sporothrix* species. Structural differences in the cell wall of rhamnomannan were also described, with longer side chains in less virulent strains, underscoring its role in *Sporothrix* species pathogenicity. Lozoya-Pérez et al. also reported the negative association between higher β-glucan exposure and virulence in *S. brasiliensis*, *S. schenckii* and *S. globosa* [16]. Although the cell wall proteome of *Sporothrix* species is poorly understood, several studies have identified adhesins, such as the 70 kDa glycoprotein (Gp70), that mediate binding to extracellular matrix (ECM) components like fibronectin and laminin [12,18,19]. Gp70 is present in *S. brasiliensis*, *S. schenckii*, and *S. globosa* [20,21], whose expression has been associated with virulence. This glycosylated cell wall protein plays a dual role in *Sporothrix* pathogenicity: it is essential for adhesion but also makes the fungus more visible to the immune system. High virulence strains are associated with Gp70 downregulation, suggesting that immune evasion through reduced expression of this surface antigen contributes to more severe or persistent infections [22]. Additionally, Pap1 (Peptidorhamnomannan-associated protein 1) plays a critical role in host–pathogen interaction by mediating adhesion to type I and II collagen, laminin, fibronectin, elastin, and fibrinogen of the ECM, facilitating fungal adherence to host tissues. Research using *Galleria mellonella* larvae revealed that challenges with Pap1 before infection protected animals from a lethal challenge of *S. schenckii* or with yeast pretreated with anti-Pap1 antibodies [23]. These data emphasize the immunogenic potential of Pap1. *Sporothrix* species cell wall polysaccharides and glycoproteins are also modified with *N*-linked and/or *O*-linked glycans. These modifications are now known to be functionally associated with fungal pathogenesis and host interaction, which, among others, seems to involve alterations in the exposure of chitin and β-glucans at the cell wall [23,24].

Melanin, a dark biopolymer integrated into the fungal cell wall, is a well-established virulence factor in *Sporothrix* species, where it plays key roles in environmental resilience, antifungal resistance, and immune evasion [25,26]. These fungi can synthesize at least three structurally distinct types of melanin—1,8-dihydroxynaphthalene (DHN)-melanin, eumelanin, and pyomelanin—through independent biosynthetic pathways [27]. Briefly, melanin is produced through the oxidative polymerization of phenolic compounds, becoming incorporated into the fungal cell wall, where it acts as a physical and chemical barrier against host antimicrobial mechanisms, namely reactive oxygen species (ROS) and nitric oxide (NO) [28,29]. Its presence also contributes to resistance against antifungal agents, such as terbinafine and amphotericin B, by reducing their accessibility to intracellular targets [26]. *In vivo* studies have demonstrated that melanin-deficient strains of *S. schenckii* markedly exhibit reduced virulence and impaired tissue invasion compared to wild-type (WT) strains [29]. In *S. globosa*, melanin has been shown to inhibit macrophage phagocytosis and suppress the host’s inflammatory response. Guan et al. found that melanin downregulates the production of pro-inflammatory cytokines, such as tumor necrosis factor alpha (TNF-α) and interleukin (IL)-6, suppresses ROS and NO synthesis, and reduces the expression of key components of the innate immune recognition system, including toll-like receptors (TLR) (e.g., TLR2 and TLR4) [30].

Fungal extracellular vesicles (EVs) are structures that facilitate intercellular communication and modulate host–pathogen interactions by transporting bioactive molecules, including proteins, lipids, polysaccharides, and nucleic acids. First described in *Cryptococcus neoformans* in 2007, EVs have been identified in several other fungal pathogens, including species of the *Sporothrix* complex [31,32]. *In vitro* and *in vivo* studies have highlighted the functional role of *S. brasiliensis* EVs in infection, demonstrating that they enhance fungal burden. In dendritic cells, these vesicles stimulate phagocytosis and increase fungal replication, while in macrophages, they upregulate MHC class II and CD86, elevate IL-6 and IL-12, and intensify fungicidal activity [33]. *In vivo*, mice pre-treated with high concentrations of EVs developed larger lesions and higher fungal loads, particularly at early stages of infection [34]. Comparative studies have revealed that *S. brasiliensis* produces EVs with a higher abundance of immunologically active components than *S. schenckii*, which can be associated with its higher pathogenic potential [33]. Proteomic analyses identified 63 proteins in EVs from *S. brasiliensis*, with 27% uncharacterized, and only 40 proteins in *S. schenckii*, of which 35% lacked functional annotation [9,34]. Most of the identified proteins are associated with stress responses, oxidation–reduction processes, and DNA metabolic activity, being essential for fungal survival under host-stress conditions. *S. brasiliensis* EVs carried immunogenic components, such as HSPs and cell wall-remodeling enzymes, while *S. schenckii* EVs were enriched in core metabolic enzymes, including glyceraldehyde-3-phosphate dehydrogenase, as well as transcription and translation factors. Only a few proteins were reported to be shared between the species, reflecting distinct virulence strategies and immune interactions. Taken together, these findings highlight EVs as critical modulators of fungal–host interactions in *Sporothrix* species and suggest that their unique molecular cargo, especially in *S. brasiliensis*, may contribute significantly to disease severity, immune evasion, and fungal persistence. Their emerging roles make them promising biomarkers and therapeutic targets in sporotrichosis.

Investigating the virulence factors of *Sporothrix* species is fundamental to elucidating the molecular mechanisms underlying sporotrichosis infection and disease progression. Characterizing these determinants of pathogenicity not only deepens our understanding of fungal–host interactions and the pathobiology of the disease but also guides the development of improved diagnostics, targeted antifungal therapies, and effective prevention and management strategies. Achieving these objectives depends critically on the continuous advancement and strategic application of molecular tools designed to study gene function and regulation in these dimorphic fungi. Genetic manipulation technologies have emerged as essential approaches for functionally validating putative virulence genes and dissecting their roles in pathogenesis. In the following section, we present the diverse genetic methodologies applied in *Sporothrix* species and how they enabled the experimental validation of key virulence factors, highlighting the impact of molecular genetics on our ability to interrogate pathogenic mechanisms at a functional level.

## 3. Molecular Tools for Genetic Manipulation in *Sporothrix* Species

Phenotypic differences in *Sporothrix* species may be linked to the expansion or contraction of specific gene families. Previous studies suggest that clinical species evolved from environmental species, with the four main clinical species (*S. brasiliensis*, *S. schenckii*, *S. globosa*, and *S. luriei*) exhibiting a high degree of endemicity [35]. Additionally, these clinical species appear to have significantly smaller genomes compared to environmental species, which correlates with a reduction in genes involved in plant decay and phytopathogenesis [36]. Such genomic differences explain the adaptive shift of *Sporothrix* species from a saprobic to a mammalian pathogenic lifestyle, marked by increased pathogenicity during evolution [36]. Investigating genomic variations between environmental and clinical strains could provide valuable insights into how phenotypic and genotypic factors drive evolution and pathogenicity in *Sporothrix* species.

Whole-genome sequencing (WGS) efforts have contributed significantly to our understanding of the genus *Sporothrix* by revealing genetic diversity, elucidating evolutionary relationships, and uncovering genes involved in virulence and host adaptation. Genomes of multiple isolates, including *S. schenckii*, *S. brasiliensis*, *S. globosa*, and *S. pallida*, have been sequenced, enabling robust comparative studies [37,38]. In 2014, Cuomo et al. conducted genome sequencing on the *S. schenckii* strain ATCC 58251, estimating the genome size at 32.23 Mb, with a GC content of 55.2% [39]. However, a separate study reported the genome sizes of *S. schenckii* (strain 1099-18) and *S. brasiliensis* (strain 5110) as 32.4 and 33.2 Mb, respectively [40]. Huang et al. further sequenced two clinical isolates of *S. globosa*, including one from a patient with therapeutic failure, reporting genome sizes of 33.47 and 33.49 Mb, respectively [38]. A comparative analysis of genomic data from 16 *Sporothrix* strains, focusing on pathogenicity, revealed that genome assembly sizes range from 33 to 44 Mb. The largest genome was identified in *S. pallida*, followed by *S. globosa*, *S. brasiliensis* and *S. schenckii* [36].

Regarding the ploidy state of the species within this complex, Torres-Guerrero initially described *S. schenckii* as diploid, based on DNA content analysis (diphenylamine method), survival to ultraviolet irradiation (UV), chemical mutagenesis, and induction of mitotic recombination [41]. Our group, by flow cytometry analyses, showed that the DNA content of resting cells matched the genome size determined by the WGS. Furthermore, our next-generation sequencing data analysis revealed a monomorphic position at each locus consistent with a haploid allele composition. Overall, these findings supported a haploid state of the *S. schenckii* analyzed strains, a conclusion reinforced by subsequent studies [28,42]. We have also demonstrated a haploid state in multiple strains of *S. brasiliensis*, *S. globosa*, and *S. pallida* [42]. Taken together, these results suggest that haploidy predominates in the genus.

Despite significant advancements in understanding the genome architecture and biological mechanisms governing the development and function of the *S. schenckii* complex, forward genetic studies remain limited. Establishing clear links between genes, their expression, and their function is critical for characterizing sporotrichosis pathophysiology, developing effective therapies, predicting clinical outcomes, and explaining the enhanced virulence of *S. brasiliensis*. Although recent developments, such as the clustered regularly interspaced short palindromic repeats (CRISPR)-associated protein (Cas) gene editing, *Agrobacterium tumefaciens*-mediated transformation (ATMT), and the generation of Δ*ku80* mutant strains, represent meaningful progress [28,43], the overall toolkit for genetic manipulation remains limited. Continued innovation in genetic manipulation strategies is urgently needed to advance *Sporothrix* functional genomics [44]. A comparative summary of the current genetic tools available for *Sporothrix* species is presented in Table 1 and described in the following sections.

### 3.1. Random Mutagenesis

Classic techniques such as chemical- and UV-induced random mutagenesis provide an easy and efficient strategy to generate mutations selectable by specific traits based on selection/identification methods. These techniques have the potential to generate both gain- and loss-of-function mutations, forming the basis for forward genetic screening [45]. Chemical mutagens take advantage of chemical compounds, such as nitrous acid and N-methyl-N′-nitro-N-nitrosoguanidine (MNNG), to introduce nucleotide substitutions or small deletions. In UV mutagenesis, the UV radiation causes DNA lesions directly or via ROS production, which further damages the DNA structure and amplifies mutation rates. Since these methods induce mutations through chemical or photonic damage, the alterations tend to occur randomly across the genome, where sites prone to replication stress may exhibit elevated mutation frequencies [45].

Torres-Guerreiro and Arenas-Lopez pioneered *S. schenckii* morphogenesis studies by attempting to obtain mutants using UV-induced random mutagenesis [46]. In this study, two different Mexican human clinical isolates (MP101 and MP102) were used as parental strains to obtain mutants with morphological differences. Exposure to UV doses of 300–1200 ergs/mm^2^ yielded morphological variants at frequencies from 0.0097 to 0.2, with survival rates of 38–64%. Interestingly, the reproducibility of the mutant phenotype remains unclear, with pleiotropic traits observed, such as altered hyphal morphology, branching, septation, and possible cell wall defects [46]. The authors also evaluated the mutagenic effect of 0.3 M nitrous acid, a base-deaminating agent, but observed mutation frequencies below 0.00001, indicating a markedly lower mutagenic potential than under UV exposure. One of the mutants isolated from *S. schenckii*, UVM9, showed pleiotropic traits, such as altered shape and size, a reversion frequency of 1 × 10^−5^ and being avirulent in a murine model [47]. Its altered cell morphology and the impaired polarized growth were suggested to be the result of mutation in any of the tubulin (α or β) genes [47]. However, a definitive link between the genotype and phenotype is missing and could be established by fully sequencing the DNA. Tachibana et al. applied MNNG-induced mutagenesis to strain IFM 41,598 of *S. schenckii* to assess the role of thermotolerance in visceral infection. After 1 hour (h) exposure to 0.3 mg/mL MNNG, viability dropped by 40%, yet all four selected mutants retained their ability to cause cutaneous lesions even at *inocula* as low as 10 colony-forming units (CFUs) [48].

Melanin, as already mentioned before, is a virulence factor in many major human pathogenic fungi, and therefore several efforts have been made to create mutants with impaired melanin production in *Sporothrix* [25]. Romero-Martínez et al. [29] used UV mutagenesis, as described elsewhere [46], on the EH-217 clinical strain of *S. schenckii* to generate pigment-deficient mutants, identifying two—an albino and a light reddish-brown variant [29]. These authors were the first to provide molecular evidence that *S. schenckii* synthesizes melanin via the DHN melanin pathway. This molecular manipulation also revealed that melanized cells were more resistant to oxidative killing and phagocytosis by human and murine immune cells [29,49]. In *S. globosa*, Song et al. [50] applied UV mutagenesis to investigate the immunomodulatory role of melanin. Melanin-deficient mutants were more susceptible to ROS and NO and induced higher levels of TNF-α and IL-6 in macrophages. Additionally, melanin downregulated TLR2 and TLR4 expression, thereby limiting innate immune activation [50].

Despite the importance of studying virulence factors, such as those cited above, producing auxotrophic mutants is a crucial step for advancing molecular tools for genetic research and selection experiments. Notably, Torres-Guerrero found significant difficulties in isolating these types of mutants using UV radiation or nitrous acid as mutagenic agents. In fact, only by using a two-step mutagenesis protocol with nitrous acid and UV radiation were auxotrophic mutants produced for adenine and methionine [41]. The inability to obtain auxotrophic mutants is still poorly understood, as the applied techniques are widely used to successfully obtain auxotrophic mutants in haploid organisms due to their recessive nature. Overall, the utilization of these random mutagenesis approaches, once standard, lost popularity due to challenges in correlating genotype to phenotype. However, the cost-effectiveness of WGS has renewed its value, making it a practical tool for dissecting gene function and virulence pathways in *Sporothrix* species, especially considering the haploid state of these species. Nevertheless, comprehensive transcriptomic and proteomic datasets are limited, which restricts the functional interpretation of genomic information. This highlights the need for an integrated, multi-omics approach to fully elucidate gene expression, protein regulation, and virulence mechanisms in this genus.

### 3.2. Genetic Transformation

Cell transformation involves the integration of exogenous DNA into a cell, being a pivotal technique in molecular biology, enabling functional genomics and deeper insights into microbial pathophysiology. This phenomenon was first demonstrated in 1928 by Frederick Griffith, who showed that non-virulent *Streptococcus pneumoniae* could be transformed into a virulent form through exposure to heat-killed virulent strains. Since then, transformation methodologies have been extensively refined, broadening their trans-kingdom applicability [51]. However, the genetic transformation of *Sporothrix* species remains particularly challenging due to their complex and variable cell wall structures and unique genomic features, which often result in low transformation efficiency and unpredictable DNA integration. Additionally, the chemical composition of the fungal cell wall can vary significantly between species, strains, and even under different environmental or growth conditions, further posing challenges to establishing standardized transformation protocols for *Sporothrix* species [28,43,52].

#### 3.2.1. Protoplast-Mediated Transformation

Protoplast-mediated transformation (PMT) is a widely used technique for genetic manipulation of filamentous fungi. This method involves enzymatic degradation of the fungal cell wall to produce protoplasts, which are then exposed to exogenous DNA under controlled osmotic conditions [53]. Due to their increased sensitivity to osmotic pressure and environmental stresses, protoplast preparation requires isotonic conditions maintained with specific salts or sugars to prevent cell lysis throughout the transformation procedure. Several factors govern the efficiency of protoplast generation and transformation, including the type and concentrations of enzymes, choice of osmotic stabilizers, digestion time and incubation temperature, fungal growth phase and mycelial age [53]. Enzymatic cocktails often contain β-glucanases, cellulases, proteases, chitinases, and α-(1,3)-glucanases, among other enzymes, for their ability to degrade diverse fungal cell wall polymers. Osmotic stabilizers like sorbitol, potassium chloride, and sodium chloride are commonly used to prevent fungal cell membrane rupture during transformation [28,53]. Polyethylene glycol (PEG) facilitates protoplast transformation by transiently enhancing the uptake of exogenous DNA into fungal cells. As an amphiphilic molecule, PEG stabilizes the membrane structure, increases membrane permeability, and promotes recovery after transformation. PEG variants commonly used include PEG 3350, PEG 6000, and PEG 8000. Higher molecular weight PEG variants increase the stability of the formed structures and reduce their toxicity; however, they significantly decrease membrane permeability. PEG is a key element in the transformation protocols, and the choice of its molecular weight directly influences the efficiency of transformation. Therefore, the choice of PEG must be carefully optimized for each fungal species to achieve the best results [53].

Rodriguez-Caban et al. developed a PMT protocol for the yeast-phase *S. schenckii*, adapting methods initially designed for *Ophiostoma* species [14]. Actively growing yeast cells were pre-treated with β-mercaptoethanol (25 mM; 1 h), which reduces disulfide bonds in cell wall proteins, thereby increasing their sensitivity to enzymatic degradation. Following the thiol step, protoplast release was induced using a mixture of β-1,3-glucanase enzymes (Glucanex^®^; 10 mg/mL, 2 h, 25 °C) in MgSO_4_ solution (1 M). In addition, sorbitol (1 M) was used to maintain protoplast integrity. For DNA transformation, approximately 10^8^ protoplasts were subjected to a PEG3350/CaCl_2_-mediated genetic transformation system. A total of 10 μg of DNA (pSD2G; plasmid-based vector) was mixed with denatured salmon sperm DNA (0.4 mg/mL). Protoplast recovery was carried out in M medium (1 M sorbitol), and transformants were selected using geneticin (500 μg/mL) as a selection marker. While this protocol enabled the genetic transformation of *S. schenckii*, the transformation efficiency was relatively low, yielding approximately 21–24 transformants per µg of DNA [14].

PEG-mediated DNA transformation of protoplasts using PEG8000 and sorbitol as the osmotic stabilizer has been applied by several authors. This protocol enabled the incorporation of DNA fragments containing either hygromycin B phosphotransferase gene (*hph*), which confers resistance to hygromycin B, or nourseothricin N-acetyl transferase (*nat*) into the *Sporothrix* genome, which are commonly used as selectable marks in genetic transformation of *Sporothrix* [28,43,54]. Unlike the protocol developed by Rodriguez-Caban et al. [14], which used the yeast phase, this method was specifically optimized for the filamentous form of the fungus [28]. Mycelia were pre-treated with Dithiothreitol (DTT; 10 mM) in citrate/phosphate buffer (90 mM; pH 7.3; 1 h; 25 °C) to reduce disulfide bridges (similar effect to β-mercaptoethanol). Protoplast release was achieved through enzymatic digestion of the cell wall using a mixture of Yatalase and lysing enzymes from *Trichoderma harzianum* for 90 min. For transformation, 5 × 10^5^ to 2 × 10^6^ protoplasts were suspended in a solution containing 50 mM CaCl_2_, 0.6 M KCl, 0.1 M Tris/HCl, pH 7.5, mixed with 25% PEG 8000 and 1.5 µg of DNA. After 25 min of incubation, additional PEG was added, and protoplasts were allowed to recover overnight in Sabouraud medium supplemented with 1.2 M sorbitol (pH 4.5) [28]. Transformants were selected using nourseothricin (80 µg/mL) or hygromycin B (180 µg/mL) as selection markers. This procedure was developed for CRISPR/Cas9 genome editing, and therefore, no transformation efficiency was reported.

**Figure 1:**
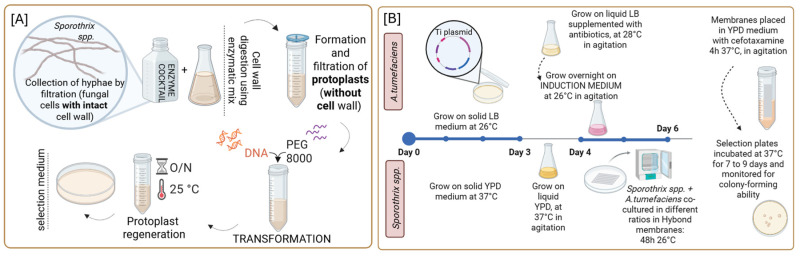
Schematic overview of transformation methods used in *Sporothrix* species. (**A**) Polyethylene glycol (PEG)-mediated protoplast transformation (PMT) begins with the collection of hyphae from young cultures, followed by enzymatic digestion of the cell wall to release hyphal protoplasts. Intact protoplasts are then separated by filtration. For transformation, the desired DNA is mixed with calcium ions and PEG, which facilitate the uptake of exogenous genetic material. Protoplast regeneration is typically performed overnight at 25 °C, allowing cell wall reformation and recovery prior to selection. Transformed cells are subsequently plated onto selective media to distinguish successful transformants from non-transformed cells. Notably, filamentous fungi show higher transformation efficiency when protoplasts are derived from actively growing mycelium, making PMT a robust and reliable method for stable genetic modification. (**B**) *Agrobacterium tumefaciens*-mediated transformation (ATMT) is a widely used method for introducing foreign DNA into fungal genomes. It begins with the construction of a binary vector carrying the genes of interest, which is introduced into *A. tumefaciens*, usually via electroporation or chemical transformation. The bacterium is then co-cultivated with fungal yeast cells under conditions that promote T-DNA transfer. After co-cultivation, fungal cells are transferred to a selective medium containing appropriate antibiotics to eliminate *Agrobacterium* and identify fungal transformants.

Protoplast-mediated transformation is a versatile and effective method for fungal genetic engineering. Successful transformation depends on the fine-tuning of enzymatic treatments, osmotic conditions, PEG properties, and the fungal growth phase. Case studies in *Sporothrix* species highlight the adaptability of PMT (Figure 1A) across different fungal morphologies and its compatibility with advanced genome manipulation tools.

#### 3.2.2. Agrobacterium Tumefaciens-Mediated Transformation (ATMT)

*Agrobacterium tumefaciens* is a Gram-negative soil bacterium best known for its natural capacity to transfer T-DNA from its tumor-inducing (Ti) plasmid into plant host genomes, leading to crown gall disease. This unique DNA transfer system has been repurposed as a genetic transformation tool across kingdoms, including fungi [55]. A key advantage of ATMT is its ability to genetically manipulate intact fungal cells, including conidia and mycelia, without requiring protoplast generation [52]. Following the demonstration by the Hooykaas group of T-DNA transfer to *Saccharomyces cerevisiae*, ATMT has been widely applied in fungal genetics to investigate traits such as virulence, sporulation, and antimicrobial resistance, among others [56].

The genetic background of *A. tumefaciens* is known to contribute to the efficiency of the ATMT system, as the bacterium’s ability to recognize and bind to the host surface depends on genes encoded within its genome. In a seminal study, *A. tumefaciens* strains LBA4404, EHA105, and AGL-1, each harboring the binary vector pBHt1 carrying the *hph* gene, were used to transform a clinical isolate of *S. schenckii* from China [52]. The transformation protocol developed was as follows: *A. tumefaciens* strains were cultured overnight in LB liquid medium (20 µg/mL rifampicin and 100 µg/mL kanamycin; 28 °C) until reaching an optical density (OD_600_) of 0.6–0.8. Washed cells were then transferred to induction medium (IM) composed of K-buffer, MN buffer, 1% CaCl_2_·2H_2_O, 0.01% FeSO_4_, 20% NH_4_NO_3_, 50% glycerol, 1 M MES (pH 5.5), and 20% glucose and incubated for 8 h at 28 °C for induction of the virulence genes. Meanwhile, *S. schenckii* was cultured on potato dextrose agar (PDA) for 7 days at 25 °C to induce sporulation. Conidia were harvested and adjusted to a final concentration of 5 × 10^6^ conidia/mL in IM. For the co-cultivation step, *A. tumefaciens* cells were mixed with an equal volume of fungal conidial suspension on solid IM plates supplemented with acetosyringone to promote T-DNA transfer. All three bacterial strains yielded transformants, though *A. tumefaciens* AGL-1 gave a higher transformation efficiency, achieving over 600 transformants per 10^6^ conidia, thereby identifying it as the most effective strain for this system [52]. Building on these findings, Lozoya-Pérez et al. [54] further refined the ATMT system using *A. tumefaciens* AGL-1 carrying pBGgHg, a vector encoding *hph* and a green fluorescent protein (GFP). The transformation was performed on the WT *S. schenckii* 1099-18 (ATCC MYA 4821). A reduced induction time of 4.5 h with IM yielded a similar transformation efficiency to previous protocols, producing approximately 722 ± 48 transformants per 10^6^ conidia [54]. In 2023, the ATMT platform was successfully extended to *S. brasiliensis* [43]. Multiple strains of *A. tumefaciens* (AGL-1, EHA105, LBA1100) carrying the *hph* marker were tested at varying fungal-to-bacterial ratios (1:1 and 1:2) and incubation durations (24, 48, 72 h). The *S. brasiliensis* yeast cell concentration was standardized to 1 × 10^8^ cells/mL. Among the tested conditions, the *A. tumefaciens* AGL-1 co-cultured at a 2:1 ratio for 48–72 h at 26 °C produced the highest transformation efficiency [43]. Real-time PCR analysis of transformants confirmed stable genomic integration of a single T-DNA copy. All these data confirmed that the ATMT system (Figure 1B) is an effective method for transforming *Sporothrix* species, although it is limited by the fact that integration occurs at random genomic locations.

### 3.3. Gene Silencing

Gene silencing techniques have emerged as vital tools to elucidate the molecular mechanisms governing the pathogenicity of several fungal pathogens, including those of *Sporothrix* species. Early work primarily focused on *S. schenckii*, where RNA interference (RNAi) was employed to investigate the function of key virulence-associated genes. A pioneer study by Rodriguez-Caban et al. on *S. schenckii* confirmed the presence of core RNAi machinery through the identification of a Dicer-1 homolog, laying the groundwork for functional gene silencing experiments [14]. Subsequent functional analyses exploited RNAi to silence genes implicated in key pathogenic processes. One of the earliest targets was the calcium/calmodulin kinase I (*SSCMK1*) gene [14]. These authors used pSD2G [57] and the protoplast transformation system to express 405 bp (3′ region) and 432 bp (5′ region) fragments of the *SSCMK1* gene. The isolated silenced mutantes exhibited an approximately 60% reduction in SSCMK1 gene expression. This silencing system showed the relevance of this gene for thermotolerance and interaction with HSP90 (two-hybrid screening), two critical factors for fungal survival and adaptation to mammalian body temperature [14]. Although orthologs of this kinase are present in *S. brasiliensis*, their precise functional roles remain to be elucidated. Complementing this pathway, DRK1 (Dimorphism-Regulating Kinase 1), a hybrid histidine kinase, acts as a master regulator of dimorphism and virulence. The *SsDRK1* gene in *S. schenckii* is highly expressed in the yeast phase, and its silencing leads to defects in cell wall synthesis, abnormal morphogenesis, reduced growth in both yeast and mycelial forms, and attenuated virulence in infection models [58,59]. These findings position DRK1 as a conserved and critical regulator in fungal dimorphism across thermally dimorphic pathogens.

Lozoya-Pérez et al. further exploited this tool and used ATMT to target the *OCH1* gene, involved in *N*-linked glycosylation, a process critical for cell wall integrity and immune evasion [60]. These authors designed an RNA hairpin structure composed of a 444 bp hairpin stem of *OCH1* (from position +54 to +497). The sense and antisense sequences flanked a spacer region consisting of an intron from the *cutinase* gene of the rice blast fungus, *Magnaporthe oryzae* [61]. With this strategy, the authors were able to reduce *OCH1* expression in *S. schenckii* by 43–99%. In addition, silencing *OCH1* in *S. schenckii* revealed alterations in cell wall composition, which correlated with reduced virulence and impaired interaction with host immune cells, thus emphasizing the role of glycosylation pathways in pathogenesis. Similarly, RNAi targeting the *ROT2* gene, a crucial enzyme for the maturation of *N*-linked glycan precursors, which encodes the catalytic subunit of endoplasmic reticulum α-glucosidase II, was also applied using the same strategy [24].

*Gp70* gene silencing in *S. schenckii* revealed its critical role in fungal adhesion, virulence, and modulation of host immune responses, underscoring the importance of this glycoprotein in pathogenesis [20]. López-Ramírez et al. used the same strategy as described above but reduced the hairpin stem to a 295 bp fragment targeting *GP70*, without affecting the silencing efficiency [20]. The results demonstrated that different levels of Gp70 expression modulated cell wall composition, namely reduced mannose, rhamnose, and protein levels, with increased β-1,3-glucan contents. The adhesin and 3-carboxy-cis, cis-muconate cyclase properties of Gp70 highlight the versatility of this protein in the fungus-host interaction. Pap1 is a recently characterized moonlighting protein found in the cell wall of *S. schenckii*, bound to peptidorhamnomannan (PRM) [23]. Given the limited knowledge of the *N*-linked glycosylation pathway in *Sporothrix* species, López-Ramírez et al. investigated the role of *ROT2*, where they generated *ROT2*-silenced strains displaying intermediate to high silencing levels, ranging from 59.3 ± 10.6% to 99.6 ± 0.1% [24]. Silencing led to the accumulation of the immature glycan core Glc_2_Man_9_GlcNAc_2_ and a concomitant reduction in total *N*-linked glycan content in the fungal cell wall. A compensatory increase in *O*-linked glycan synthesis was described, proportional to the degree of *ROT2* silencing.

In a seminal work, Padró-Villegas et al. extended this research to *S. brasiliensis* [62]. Silencing *Gp70* in *S. brasiliensis* involved a similar strategy as described above, with a 295 bp fragment of the 5′ end of the *GP70*, and transformation was *Agrobacterium*-mediated. The *Gp70* silencing in *S. brasiliensis* resulted in morphological changes, including smaller, round yeast cells prone to aggregation. Cell wall analyses revealed increased β-1,3-glucan exposure and diminished *N*-linked glycan content, which likely contributed to altered immune recognition. Furthermore, mutants showed decreased enzymatic activity of 3-carboxy-cis, cis-muconate cyclase, impaired adhesion to laminin and fibronectin, and modified cytokine responses upon interaction with human peripheral blood mononuclear cells. These alterations culminated in attenuated virulence in the *G. mellonella* infection model [62]. This landmark study represents the first RNAi application in *S. brasiliensis* and establishes a functional link between Gp70 and fungal pathogenicity.

RNAi-based gene silencing has proven to be an effective and relatively accessible tool for functional studies in *Sporothrix*, particularly given the early-stage development of advanced genetic tools in this genus. Its main strengths lie in the ability to achieve graded knockdown of gene expression without requiring gene disruption, which is especially advantageous when targeting essential genes. However, this approach presents several limitations, including variable silencing efficiency, the potential for off-target effects, and challenges in achieving complete gene suppression. Furthermore, the construction of hairpin RNA vectors and reliance on transformation systems such as ATMT or PMT may restrict their widespread application, especially in strains or laboratories with limited genetic tools. Despite these limitations, RNAi remains a valuable strategy for target validation and functional genomics, particularly in *Sporothrix*, where CRISPR-based editing is only beginning to emerge. The growing number of successful RNAi applications in both *S. schenckii* and *S. brasiliensis* underline its potential as a bridge between classical and modern molecular fungal biology.

### 3.4. Gene Editing

Gene editing enables precise modifications to the genome sequences, offering a powerful tool for understanding and manipulating genetic functions. Among these technologies, the CRISPR system has emerged as one of the most efficient gene-editing methods. Originally discovered as an endogenous immune defense mechanism of bacteria and archaea against invading viruses [63], the CRISPR system has since been repurposed as a powerful biotechnology tool that enables gene replacement, deletion, and insertion in both eukaryotic and prokaryotic organisms. To date, three CRISPR system types (I-III) have been identified, with type II being the most widely used and well-characterized. This type relies on a single protein nuclease for DNA cleavage. First introduced in *Saccharomyces cerevisiae* [64], the CRISPR/Cas system has revolutionized genetic studies in fungi, including filamentous fungi [65]. The selection of transformants often relies on positive selection markers, namely resistance markers or nutrient deficiency genes, though options for filamentous fungi remain limited [65].

CRISPR-based tools have emerged as pivotal in the targeted genetic manipulation of pathogenic *Sporothrix* species, *S. brasiliensis* and *S. schenckii*, particularly in understanding their virulence mechanisms [28]. The internalization of CRISPR/Cas9 protein complexes assembled with guide RNAs (gRNAs) via PEG-mediated protoplast transformation enables precise gene editing. This was exemplified by the deletion of the *pks1* gene, which encodes 1,3,6,8-tetrahydroxynaphthalene synthase, in *Sporothrix* species [28]. The gene-editing strategy relied on the integration of a cassette carrying either nourseothricin or hygromycin B resistance markers, each flanked by approximately 850 bp of homology region positioned upstream and downstream of the Cas9 enzyme cleavage sites at the targeted gene. For this purpose, the EnGen Spy Cas9 NLS was guided with two different gRNAs, locating the Cas9 at the starting and ending regions of the open reading frame. Hatinguais and colleagues increased the efficiency of cassette integration through homologous recombination by using strains null for *KU80* homolog (SPBR_02356 and SPSK_07043). The deletion of *KU80* impairs non-homologous end-joining (NHEJ) DNA repair pathway, thereby favoring homologous recombination, as has been described for other systems [66]. The full gene-editing protocol begins with the cultivation of *Sporothrix* species in Sabouraud dextrose broth at 25 °C to obtain actively growing mycelia. The protoplasts are generated by enzymatic digestion using Yatalase and Lysing Enzymes from *Trichoderma harzianum* in a maleic acid-based buffer. These protoplasts are then incubated with preassembled Cas9/gRNA ribonucleoprotein complexes along with linearized donor DNA. Transformation is carried out using PEG 8000 in a Tris-HCl buffer supplemented with CaCl_2_. Regeneration is performed in sorbitol-containing Sabouraud medium, and transformants are selected using either nourseothricin or hygromycin B resistance.

These seminal advances address the historical lack of molecular tools for reverse genetics in these fungi, representing a critical step towards understanding the virulence mechanisms of *Sporothrix* species and providing a foundation for further functional studies. This technique, when fully applied in *Sporothrix* studies, will allow a remarkable evolution in our understanding of these pathogens and their associated diseases.

### 3.5. Heterologous Expression for Functional Gene Analysis

Filamentous fungi serve as versatile platforms for heterologous gene expression, offering valuable insights into gene function and facilitating the elucidation of biosynthetic pathways. Their capacity to accommodate and express genes from diverse organisms makes them indispensable tools in functional genomics and natural product research. The use of forward genetics approaches, particularly through functional complementation using mutant libraries such as the *S. cerevisiae* Euroscarf collection or *Candida* species mutants, is highly valuable for elucidating gene function. Currently, the repertoire of tools available for understanding gene function and relevance in *Sporothrix* is limited. In pathogenic fungi such as *S. schenckii*, heterologous expression has been instrumental in characterizing genes involved in glycosylation pathways. For instance, a genome-wide screening identified several *S. schenckii* genes belonging to glycosyl hydrolase families 47 and 63. These genes were individually expressed in *C. albicans* null mutants lacking specific glycosidase genes. Restoration of *N*-linked glycosylation-associated mutant phenotypes confirmed their functional role in glycan maturation [67]. Furthermore, heterologous expression of *Sporothrix schenckii KTR4* and *KTR5*, members of the *MNT1/KRE2* gene family, in *Candida albicans* null mutants resulted in functional complementation of *N*-linked glycosylation defects, confirming the roles of these *Sporothrix* genes as Golgi-resident mannosyltransferases [68]. Similarly, the isolation and characterization of the *S. schenckii ROT2* gene is crucial for *N*-linked glycosylation. Robledo-Ortiz et al. demonstrated that this gene could partially restore the phenotype in an *S. cerevisiae rot2*Δ mutant, underscoring its functional conservation and potential for studying glycosylation processes in pathogenic fungi [69]. Further, the *GFA1* gene from *S. schenckii*, encoding glucosamine-6-phosphate synthase—a key enzyme in amino sugar biosynthesis—was successfully cloned and expressed in an *S. cerevisiae* gfa1Δ mutant. The heterologously expressed gene not only restored prototrophy but also resulted in enzyme activity with improved stability and resistance to product inhibition, suggesting biotechnological advantages [70].

The potential of this genetic approach was also demonstrated through the expression of two cell wall proteins from *S. schenckii*, Hsp60 and PRM-associated protein 1 (Pap1), in *E. coli*. These proteins, components of the PRM complex, exhibited adhesive properties to ECM proteins and activated immune responses in invertebrate models, positioning them as promising candidates for immunological studies [23]. Tavares et al. also developed a plasmid toolkit for *Sporothrix* species that enables fusion of endogenous genes to fluorescent markers such as sGFP and mCherry. The toolkit allowed precise subcellular localization studies, exemplified by the successful nuclear targeting of a histone H2A fusion protein under the control of the pGAPDH promoter, in both yeast and mycelial forms of the fungus [43]. Similarly, Hatinguais et al. [28] demonstrated that PMT is a reliable method for expressing exogenous genes, such as a synthetic, red-shifted luciferase. Combined with CRISPR/Cas9 technology, this system provided a robust platform for investigating fungal viability under ROS stress and represents a promising approach for in vivo virulence studies [28].

Although heterologous expression has proven to be a valuable tool for elucidating gene function in *Sporothrix* species, functional conservation across hosts does not always guarantee accurate phenotypic outcomes. Differences in codon usage between the donor and host organism can reduce protein accumulation and affect protein folding and stability, thereby influencing the resulting phenotypes. Additionally, proteins expressed in a heterologous system may acquire host-dependent additional or moonlighting functions that are absent in the native context, further complicating functional interpretation. Despite these limitations, heterologous expression remains a critical tool in fungal functional genomics, particularly given the technical constraints associated with genetic manipulation in *Sporothrix* spp. It has provided insights into glycosylation pathways, cell wall protein function, subcellular localization, responses to stress, and the development of versatile platforms for immunological and in vivo virulence studies.

## 4. Future Perspectives

The ongoing development of molecular tools for *Sporothrix* species marks a pivotal step toward unraveling the genetic basis of virulence, host adaptation, and antifungal resistance in this genus. Despite notable advancements—such as the establishment of PEG-mediated protoplast transformation, ATMT and CRISPR/Cas9-based gene editing—the toolkit for efficient and targeted genetic manipulation remains relatively limited compared to model fungal pathogens.

One of the most transformative recent breakthroughs has been the implementation of a CRISPR/Cas9 system optimized for *S. brasiliensis* using PEG-mediated transformation. This system enabled precise deletion of the *pks1* gene and demonstrated enhanced editing efficiency in ∆*ku80* mutants, which exhibit impaired NHEJ and improved homologous recombination rates [28]. Building upon this foundation, future studies should expand gene targeting strategies to include genes involved in immune evasion, biofilm formation, and metabolic adaptation, which are central to the success of pathogens in both environmental and host-associated niches. Given the demonstrated utility of fluorescently or luminescently tagged strains [28,43,54] the next logical step involves leveraging these strains in live imaging and host–pathogen interaction models. Tagging known or suspected virulence genes with fluorescent markers in vivo will facilitate dynamic studies of fungal dissemination, tissue tropism, and immune evasion in real time. Another promising avenue is the integration of transcriptomic and proteomic data with reverse genetics. In fact, coupling CRISPR/Cas9 mutagenesis with RNA-Seq or proteome-wide screens can help map regulatory networks and uncover condition-specific gene functions. Furthermore, introducing CRISPR interference (CRISPRi) or activation (CRISPRa) systems, coupled with Tet-On/Tet-Off regulation, would allow fine-tuned, reversible control of gene expression, critical for essential genes or subtle phenotypic traits [71]. Moreover, the development of self-replicating plasmid systems will be crucial, as they have proven effective in other fungal pathogens for increasing transformation efficiency and enabling precise genetic manipulation. These plasmids, particularly in their linear form, have demonstrated the ability to increase the yield of autonomous transformants and support stable gene expression without chromosomal integration. Applying such technologies to *Sporothrix* could overcome current limitations in genetic transformation and accelerate functional genomics studies. Future research should focus on identifying or engineering replication origins compatible with *Sporothrix* species, optimizing transformation protocols, and validating the use of episomal vectors in these fungi.

In summary, the field of *Sporothrix* research is undergoing a critical transition, from largely descriptive studies to a functional genomics *era* driven by advanced molecular tools. Collectively, the molecular toolbox for *Sporothrix* species now includes transformation systems (protoplast-mediated and *Agrobacterium tumefaciens*-mediated), gene silencing (RNAi), CRISPR/Cas9-based gene editing, heterologous expression platforms, and fluorescent/luminescent reporter systems. While each tool has typically been used in isolation to address specific questions, its true potential lies in their integration. For instance, CRISPR-mediated gene disruption can be combined with fluorescent tagging to study dynamic localization, while RNAi or CRISPRi can enable tunable knockdowns in essential genes. Heterologous expression further facilitates functional validation in genetically tractable hosts. These complementary approaches allow researchers to dissect complex traits—such as virulence, immune evasion, or metabolic adaptation—through direct manipulation and surrogate modeling. A coherent strategy that harnesses multiple tools will accelerate the transition from gene identification to mechanistic understanding in this emerging pathogen.

## Figures and Tables

**Table 1:** Comparative overview of genetic manipulation tools applied to *Sporothrix* species (key references are described in the text section).

Genetic Tool	Mechanism	Applications	Strengths	Limitations
**Random mutagenesis (UV or chemical)**	Induces genome-wide mutations via UV light or mutagenic agents (e.g., N-methyl-N′-nitro-N-nitrosoguanidine (MNNG), nitrous acid)	Analysis of thermotolerance, morphology, melanin biosynthesis, virulence attenuation	Technically simple; suitable for gain- and loss-of-function screening	Low reproducibility; genotype–phenotype link often unclear; laborious mutant characterization
**Protoplast-mediated transformation (PMT)**	Cell wall enzymatic digestion generates protoplasts for DNA uptake via Polyethylene glycol (PEG)/CaCl_2_ transformation	Introduction of plasmids, resistance markers, and CRISPR/Cas9 components	Applicable to yeast and mycelial forms; compatible with ribonucleoprotein delivery	Low transformation efficiency: species-specific optimization required
***Agrobacterium tumefaciens-*** **mediated transformation (ATMT)**	T-DNA transfer from engineered *A. tumefaciens* into fungal genome during co-cultivation	Stable genomic integration; gene silencing via RNAi; green fluorescent protein (GFP) expression	No protoplasts required, high efficiency in hyphae and yeast cells	Integration sites are random; relies on bacterial–fungal compatibility
**RNA interference (RNAi)**	Expression of hairpin RNA triggers post-transcriptional gene silencing via the Dicer/RISC complex	Functional analysis of virulence genes (*GP70*, *OCH1*, *DRK1*, *ROT2*)	Enables partial knockdown; useful for essential genes; no genomic editing required	Variable silencing efficiency; off-target effects; transformation-dependent
**CRISPR/Cas9 gene editing**	Cas9/sgRNA complex introduces targeted Double-Strand Break; repaired by non-homologous end-joining (NHEJ) or homologous recombination with donor DNA	Targeted gene deletion (*pks1*); use of Δ*ku80* strains to enhance homologous recombination	Precise genome editing; improved efficiency in recombination-deficient strains	Dependent on protoplast transformation; early-stage adoption in *Sporothrix* spp.
**Heterologous expression**	Cloning and expression of *Sporothrix* genes in model organisms (e.g., *S. cerevisiae*, *C. albicans*, *Escherichia coli*)	Functional complementation; characterization of glycosylation enzymes, adhesins (e.g., ROT2, GFA1, Gp70, Pap1)	Circumvents low transformation rates in native host; enables protein-level studies	May lack post-translational modifications of native host; partial functional conservation

## Data Availability

No new data were created or analyzed in this study. Data sharing is not applicable to this article.

## Abbreviations

The following abbreviations are used in this manuscript:

ATMT	*Agrobacterium tumefaciens*-mediated transformation
CaM	Calmodulin
CaMKs	Calmodulin-dependent protein kinases
CFUs	Colony-forming units
CRISPR	Clustered regularly interspaced short palindromic repeats
DHN	1,8-dihydroxynaphthalene
DRK1	Dimorphism-Regulating Kinase 1
DTT	Dithiothreitol
ECM	Extracellular matrix
EVs	Extracellular vesicles
GFP	Green fluorescent protein
Gp	Glycoprotein
gRNAs	Guide RNAs
h	Hour
*hph*	Hygromycin B phosphotransferase gene
HSPs	Heat shock proteins
IL	Interleukin
IM	Induction medium
min	Minutes
MNNG	N-methyl-N′-nitro-N-nitrosoguanidine
*nat*	Nourseothricin N-acetyl transferase gene
NHEJ	Non-homologous end-joining
NO	Nitric oxide
OD	Optical density
Pap1	Peptidorhamnomannan-associated protein 1
PEG	Polyethylene glycol
PMT	Protoplast-mediated transformation
PRM	Peptidorhamnomannan
RNAi	RNA interference
ROS	Reactive oxygen species
SSCMK1	calcium/calmodulin kinase I
Ti	Tumor-inducing
TLR	Toll-like receptor
TNF-α	Tumor necrosis factor alpha
UV	Ultraviolet irradiation
WT	Wild-type
WGS	Whole-genome sequencing
